# Why do sunflowers have invisible colors?

**DOI:** 10.7554/eLife.76105

**Published:** 2022-01-21

**Authors:** Jason Laurich, Anna M O'Brien

**Affiliations:** 1 Department of Ecology and Evolutionary Biology, University of Toronto Toronto Canada

**Keywords:** sunflower, floral pigmentation, pollination, abiotic stress, adaptation, plants, Other

## Abstract

In the common sunflower, patterns of UV-absorbing pigments are controlled by a newly identified regulatory region and may be under the influence of environmental factors.

**Related research article** Todesco M, Bercovich N, Kim A, Imerovski I, Owens GL, Dorado Ruiz Ó, Holalu SV, Madilao LL, Jahani M, Légaré JS, Blackman BK, Rieseberg LH. 2022. Genetic basis and dual adaptive role of floral pigmentation in sunflowers. *eLife* **11**:e72072. doi: 10.7554/eLife.72072

We may delight at the sight of colorful flowers, but some of their beauty often remains hidden. Floral structures are typically intended for insect pollinators that can perceive UV light and therefore ‘see’ additional colors invisible to humans. The petal-like structures around the center of sunflowers, for example, appear plain yellow to our eyes but they feature a UV-absorbing base and UV-reflecting tip; this creates a ‘bullseye’ pattern that is thought to guide insects towards the flower (Figure 1A, Koski and Ashman, 2013). The size of the bullseye can vary widely between and even within sunflower species, but the genetic mechanisms that regulate this variation in UV absorbance remain unknown (Moyers et al., 2017).

**Figure 1.**
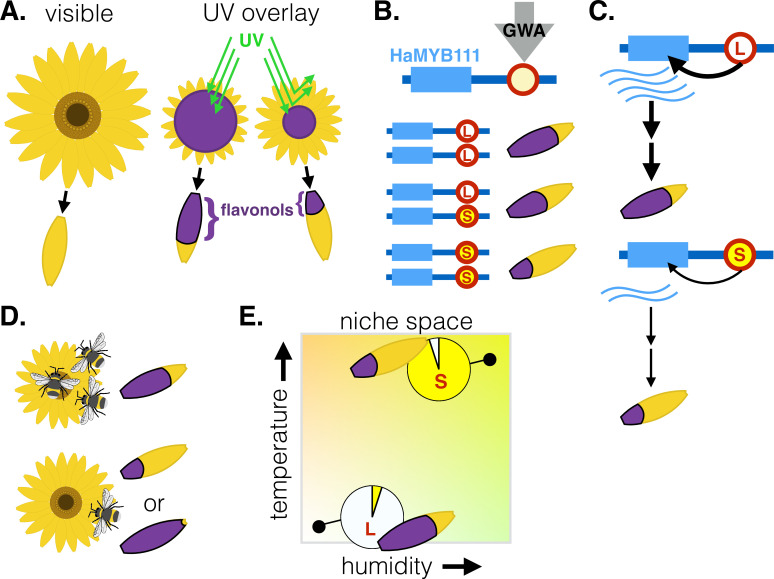
Genetic factors controlling the size of UV-absorbing patterns in the common sunflower, and their relationship with environmental factors. (**A**) To the human eye, sunflowers vary little in color, yet some parts of the flower (shown in purple) also absorb UV light (green lines) that we cannot see. These UV-absorbing regions vary in size due to differences in the distribution of flavonol pigments in modified petals called ligules. This creates a ‘bullseye’ pattern that varies in size. (**B**) Genome-wide association analysis (GWA) of parents and crosses of *Helianthus annuus* plants from various locations identified a locus (red circle) upstream of the *HaMYB111* gene which underlies variation in UV absorbance; one allele is associated with large bullseyes (L, white) and the others with small ones (S, yellow). (**C**) The large bullseye allele is associated with increased expression of *HaMYB111*, which appears to increase the deposition of UV-absorbing flavonols in the ligules compared to the small allele. (**D**) Pollinators prefer sunflowers with intermediate UV absorbance – and medium bullseyes. (**E**) Smaller UV-absorbing regions and higher frequencies of the small UV-absorbing allele are found in populations from warmer and more humid climates (top right). The graph shows theoretical populations.

As well as being thought to act as pollinator guides, the pigments that allow plants to absorb UV light and create patterns may also protect pollen from UV damage. In addition, they can alter flower temperatures by trapping heat or preventing evaporative cooling (Koski and Ashman, 2013; Koski and Ashman, 2015; Koski et al., 2020). Yet, in most plants, it is unclear which of these effects drive the evolution of UV absorbance. Now, in eLife, Marco Todesco, Loren Rieseberg and colleagues at the University of British Columbia and the University of California, Berkeley, report on the genetic factors underlying variation in the size of sunflowers’ bullseyes, how these structures affect pollination, and how the environment may have altered their prevalence (Todesco et al., 2022).

The team first focused on the common sunflower (*Helianthus annuus*), which is distributed across much of North America and therefore an excellent system to understand how UV absorbance evolves. Seeds were collected from across the species’ living range and plants from various locations were bred together. This allowed Todesco et al. to conduct a genome wide association analysis to pinpoint the genetic variation associated with the size of the bullseye.

The work highlighted variation at a small region upstream from the *HaMYB111* gene, which was responsible for 60% of the observed variation in bullseye size. One allele was linked to low expression of *HaMYB111* and a pigment distribution that creates small bullseyes, while the other was linked to large bullseyes (Figure 1B). Flowers with smaller bullseyes, for example, had two copies of the ‘small pigment’ allele and expressed lower levels of *HaMYB111* during development, when the pattern of UV pigments is established.

To understand the role of *HaMYB111*, Todesco et al. harnessed another plant (*Arabidopsis thaliana*) in which more genetic tools are available. They used this model to demonstrate that *A. thaliana*’s version of *HaMYB111* controls the expression of UV-absorbing pigments. In *H. annuus*, the region identified by the team likely alters the transcription of *HaMYB111*, therefore regulating the production of UV-absorbing pigments and the development of bullseyes (Figure 1C).

Further experiments revealed that insect pollinators preferred medium bullseyes over the smallest and largest ones, but it was climatic factors that best matched range-wide variation in the size of the pattern (Figure 1D–E). For instance, plants in drier, colder sites had larger bullseyes and higher frequencies of the associated allele: this is consistent with climatic selection for UV absorbance, as pigments also promote heat retention and reduce water loss.

Finally, examining dozens of sunflower species highlighted substantial diversity in the size of the bullseyes. This suggests an ‘invisible’ evolutionary radiation, wherein sunflowers may have faced divergent local selection on UV absorbance while speciating. Whether this hidden variation across species recapitulates patterns found in common sunflowers and reflects divergence in species’ climatic niches should be further explored. Other research might also consider whether bullseyes could directly affect the formation of new sunflower species or hybrids, since differences in pigmentation can affect cross-pollination (Baack et al., 2005; Hopkins and Rausher, 2012). Further work should also address other regulatory mechanisms underpinning variation in UV-absorbance, given the team’s finding that bullseye variation in another species of North American sunflowers (*Helianthus petiolaris*) was correlated with different genetic regions.

Overall, the work by Todesco et al. showcases how biological and climatic factors interact to shape floral evolution. Wet, warm climates, for example, could lead to an evolutionary challenge: a pollinator may prefer large UV-absorbing bullseyes but these may prevent evaporative cooling mechanisms beneficial in that environment. In this context, compensatory selection could act on other traits that attract pollinators, such as the production of nectar or the release of certain volatile compounds (Rering et al., 2020). Research into these mechanisms is key as climate change may alter how pollinators and environment drive the evolution of UV absorbance by increasing warming stress, affecting interactions between plants and pollinators, and reducing insect populations (Settele et al., 2016; Rering et al., 2020). In fact, a much broader swathe of the tree of life could similarly be facing trade-offs between different types of evolutionary pressures which drive local adaptation and constrain responses to selection.
